# Hyperimmune serum containing *Histophilus somni* rHsp60, rOMP40 and *Actinobacillus pleuropneumoniae* LPS antibodies as supplementary treatment for calf respiratory diseases

**DOI:** 10.1038/s41598-025-03624-1

**Published:** 2025-05-30

**Authors:** Krzysztof Blicharski, Joanna Bajzert, Paulina Jawor, Wojciech Jachymek, Marian Kuczaj, Tadeusz Stefaniak

**Affiliations:** 1 Department of Immunology, Pathophysiology and Veterinary Preventive Medicine, Wrocław University of Environmental and Life Sciences, C.K. Norwida 31 Str., 50-375 Wrocław, Poland; 2 Department of Immunochemistry, Hirszfeld Institute of Immunology and Experimental Therapy, Polish Academy of Sciences, Weigla St. 12, 53-114 Wrocław, Poland; 3 Institute of Animal Husbandry and Breeding, Wrocław University of Environmental and Life Sciences, Chełmońskiego St. 38C-E, 51-630 Wrocław, Poland

**Keywords:** Hyperimmune serum, Bovine respiratory disease, Calf serotherapy, Subunit vaccine, Immunotherapy, Infection, Infectious diseases, Inflammation, Protein vaccines, Immunology

## Abstract

Bovine respiratory disease (BRD) in dairy heifer calves cause significant economic losses associated with treatment costs, reduced growth rate and mortality. Bovine hyperimmune serum was produced in cows hyperimmunized with three conserved antigens (*Histophilus somni* rHsp60, *H. somni* rOMP40, and *Actinobacillus pleuropneumoniae* LPS core oligosaccharide), which exhibit broad cross-reactivity with several Gram-negative bacteria. Dairy heifer calves received serum subcutaneously on the first day of treatment for BRD, as an additive to antibiotics and anti-inflammatory drugs. Health status was estimated on a modified point scale system on days 1, 7 and 14th of the treatment. The calves were divided into four groups (10 per group): two with moderate signs of BRD, one with and one without hyperimmune serum application, and two with severe signs of BRD, one receiving hyperimmune serum. At day 1,7,14 of treatment and day 180 of age, plasma fibrinogen, serum haptoglobin, serum amyloid A, IgG_1_, IgG_2_, and IgM concentrations were estimated. Harvested hyperimmune serum contained high levels of IgG_1_ and IgG_2_ antibodies against homologous antigens. The findings suggest that the supplementary use of hyperimmune serum, in combination with antibiotics and NSAIDs, may contribute to an earlier improvement and reduced severity of clinical signs in dairy heifer calves compared to treatment without serum.

**Supplementary Information:**

The online version contains supplementary material available at 10.1038/s41598-025-03624-1.

## Introduction

Bovine respiratory disease (BRD) commonly affects young dairy heifers^1^. The high incidence of respiratory tract disease in dairy heifer calves may irreversibly affect their growth and development, increase premature culling and age at first calving, and influence their further milk yield and longevity^2–4^.

The pathogenesis of BRD involves stressful conditions coupled with viral infection, resulting in suppressed immune defenses that lead to bacterial proliferation in the upper respiratory tract. Subsequently, these bacteria colonize the lower respiratory tract and cause bronchopneumonia with cranioventral distribution in the lungs. Many of the bacteria involved are commensal organisms of the upper respiratory tract that can be isolated from healthy animals^5,6^. The bacterial pathogens most commonly associated with BRD include *Pasteurella multocida*, *Histophilus somni, Mannheimia haemolytica* and *Mycoplasma bovis*^7–9^. Viral pathogens, such as bovine herpesvirus 1 (BoHV-1), parainfluenza-3 virus (PI-3V), and bovine respiratory syncytial virus (BRSV), may also be involved in disease development. Diagnosing BRD is challenging. Detection of the clinical progression of BRD may be facilitated by the presence of an inflammatory response, isolation of infectious agents, and seroconversion^10,11^.

The prevalence of bovine respiratory diseases has increased despite the use of antibiotics and vaccines^12^. One way to prevent and treat respiratory diseases is the administration of antisera. Hyperimmune serum is an important source of antibodies. It has been shown that commercial polyvalent immune serum against *Escherichia coli, Salmonella* Dublin and *S*. Typhimurium given intravenously to colostrum-deprived calves protected them against death after oral challenge with *E. coli* O78:K80 (B)^13^. The administration of hyperimmune plasma produced in cows immunized with *E. coli,* Rotavirus and Coronavirus, *Pasteurella*, bovine viral diarrhea virus (BVDV), and *Clostridium* spp. vaccines, especially in cases of passive transfer failure in calves, is advisable for the prophylaxis and treatment of neonatal calf diarrhea and could reduce the use of antibiotics and the risk of antibiotic resistance in farm animals^14^. Haralambiev et al.^15^ showed that intratracheal (3–5 mL) or intramuscular (20 mL) administration of hyperimmune serum containing antibodies against PI-3V and bovine adenovirus to calves during outbreaks of respiratory diseases reduced the incidence of the disease by approximately five times.

Our previous studies indicated that animals hyperimmunized with whole cells or outer membrane antigens of *Histophilus somni* developed antibodies that reacted with the majority of representatives of the *Pasteurellaceae* and *Enterobacteriaceae* families^16–18^. Based on previous findings, we selected and produced two recombinant protein antigens (*H. somni* rHsp60 and *H. somni* rOMP40)^19–21^ that showed high amino acid sequence homology with similar proteins of the *Pasteurellaceae* and *Enterobacteriaceae* families. Antibodies against these proteins demonstrated cross-reactivity with Gram-negative bacteria^19,20^. These selected antigens were conjugated with *Actinobacillus pleuropneumoniae* LPS core carbohydrate. The LPS core carbohydrates expressed by *A. pleuropneumoniae* are similar to antigens expressed by other mucosal pathogens, such as *P. multocida*^22^. The obtained antigenic composition contained only a small component of the whole LPS, in contrast to a vaccine based on Gram-negative bacteria^23^.

The aim of this study was to produce bovine hyperimmune serum containing antibodies against selected conserved antigens of Gram-negative bacteria that present broad cross-reactivity with related antigens of many strains of *Pasteurellaceae* and *Enterobacteriaceae* families, and evaluate the effect of supplementary application in the treatment of respiratory tract infections in dairy heifer calves.

## Materials and methods

### Animals

The study was performed on Polish Red Holstein–Friesian cows and heifer calves originating from three dairy farms (A with 170 cows; B with 220 cows; and C with 330 cows) owned by one company. The adult cows in the herds were vaccinated against BoHV-1 (IBR-Marker Inactivatum, Zoetis, USA), BVDV (Bovilis BVD, MSD Animal Health, USA), and Rotavec Corona (Bovilis Rotavec Corona, MSD Animal Health) before calving.

### Production of hyperimmune serum

Five cows (no. I-V) from the C farm were selected for immunization. The last vaccination of these cows against BoHV-1 and BVDV occurred 10 months before the beginning of immunization. Cows were immunized subcutaneously (s.c.) in the dewlap region six times with the experimental vaccine previously used in pigs^24^. The composition contained recombinant 60 kDa heat shock protein from *Histophilus somni* (*H. somni* rHsp60; 10 μg/dose; produced by Pure-Biologics Ltd. Wroclaw, Poland), recombinant 40 kDa outer membrane protein from *H. somni* (*H. somni* rOMP40; 20 μg/dose; produced by Pure Biologics Ltd. Wroclaw), as well as *A. pleuropneumoniae* LPS core oligosaccharide conjugated with *H. somni* rHsp60 (20 μg/dose) and *A. pleuropneumoniae* LPS core oligosaccharide conjugated with rOMP40 (40 μg/dose). Antigens were dissolved in 1.0 ml of saline containing 20% v/v Emulsigen® (MVP Technologies, USA).

Blood samples were collected seven times. Immediately before the first s.c. immunization (S1), then every two weeks immediately before the next s.c. immunization (S2-S5), then at the last immunization, which was three weeks after the fifth immunization (S6), and three weeks after the last immunization (S7). Before each immunization, blood samples were collected from the jugular vein using a needle (18Gx1 1/2″; BD Becton Dickinson SSC; USA) and a 2 mL tubes with K2EDTA (Profilab, Poland) and 11 mL with a clot accelerator and granule serum separator (Profilab, Poland). Blood serum (11 mL tubes) was harvested by centrifugation (15 min, 1860 × g) within 2 h of collection. The cow individual serum samples were stored at − 80 °C. The blood samples taken for the anticoagulant were used for the analysis of fibrinogen and hematocrit.

To produce serum two weeks after the 4th immunization (S5), five liters of blood was collected from each cow, and the cows were injected with the next dose of the vaccine. Three weeks after the 5th immunization (S6), five liters of blood was collected from each cow and the animals were revaccinated. Three weeks later (S7), five liters of blood was collected from each donor cow as previously described^25^. Blood was collected into separate plastic disinfected buckets then was pooled and manually mixed for 15 min to eliminate fibrinogen. In the laboratory, collected blood was centrifuged in room temperature for 20 min at 1100 × g to separate the blood cells from the serum, which was then collected. The obtained serum within 5 h was phenol preserved to achieve a final concentration of 0.5%. The pooled serum from the last three samplings (S5-S7) was mixed in one container, poured into 0.5 L sterile bottles, and used for the treatment of the calves.

The health of the cow was evaluated based on clinical examination. All the cows were deemed healthy at the start of the study and remained healthy throughout the study.

### Calves’ management

On all farms (A, B, and C), newborn calves were kept in individual pens located close to the calving area and housed for the first 2–3 days after birth. Up to the 3rd hour of age, the calves were fed colostrum from their dams. Neither the quality nor quantity of colostrum was measured. The calves were then moved to individual plastic calf hutches (igloo) with a hutch pen size of 2 m^2^. Until the 5th day of age, they were fed with maternal colostrum and transition milk. From the 6th to the 14th day, they were fed twice a day with milk replacer Kalbi Milch Classic (Schaumann Agri International GmbH, 20% protein, 18% fat) 6 L per day, from the 15th to the 30th day, 7 L per day. After 3 weeks, calves were moved to group pens (6–8 individuals, approximately 1.5 m^2^/head) with plastic hutches. The pens were located on a concrete floor with straw bedding. On farm A, the hutches and pen hutches were covered from three sides; on farm C, the hutch pen was not covered with a roof; and on farm B, the hutches were inside the calf barn. From the 31st to the 60th day of age, calves were fed twice a day with milk replacer, 8 L per day. From day up to eight week of age calves had also ad libitum access to muesli juniors (Vittra Müsli Junior) mixed with alfalfa hay. At age 6–8 weeks calves were transported to a common calf barn located at farm A, where they were raised to the age of 6 months. From 60 to 90 days of age, calves were fed muesli (Vittra Müsli Junior) mixed with a total mix ration (TMR from another group; see Supplementary file Table S1). After the 90th day of age, only TMR was provided. The distance to farm A from farm B was 6 km and that from farm C was 15 km. In this calf barn, calves were grouped according to age and body weight in pens of 20–25 heads and kept until the age of 6 months. Each pen has access to a paddock.

### Calf experimental design

On the farm, the clinical respiratory cases were monitored and treated by a farm veterinarian. The modified Wisconsin BRD scoring system^26^, was used for calf health scoring criteria. Modification included an increase in the threshold for scoring fever (39.4 °C) as a pathological sign and auscultation of the lungs (Table 1).

**Table 1:** Calf health scoring criteria for the evaluation of severity of clinical signs of respiratory tract disease in calves.

Parameter	Score			
	0	1	2	3
Rectal temperature (°C)	< 39.3	39.4–39.6	39.7–40.0	> 40.0
Cough	None	Induce single cough	Induced repeated coughs or occasional spontaneous cough	Repeated spontaneous coughs
Nasal discharge	Normal serous discharge	Small amount of unilateral cloudy discharge	Bilateral, cloudy or excessive mucus discharge	Copious bilateral mucopurulent discharge
Eye scores	Normal	Small amount of ocular discharge	Moderate amount of bilateral discharge	Heavy ocular discharge
Ear scores	Normal	Ear flick or head shake	Slight unilateral droop	Head tilt or bilateral droop
Lung auscultation	Normal	Bronchial	Cracles or wheezing	x

When the staff noticed any signs of respiratory disease, the calf was evaluated using a clinical evaluation score (Table 1). If the calf scored 5–10 points, it was categorized as showing moderate (M) signs, and if scored 11 or more points, it was qualified as showing severe (S) clinical signs. The sick calves were systematically allocated to either the experimental group (E, with hyperimmune serum as an additional treatment) or the control group (C, treatment without serum), using an alternating sequence based on the score of clinical signs. Finally, depending on the severity of the disease and treatment, four groups of calves (EM, ES, CM, and CE) were formed with ten calves each (total number of calves n = 40; for each group n = 10). A limited number of calves could be included in the study due to the inclusion of calves with severe BRD. Calves in the experimental and control groups were treated with florfenicol (Floron, Krka, Slovenia) 40 mg/kg body weight s.c. once and flunixin (Vetaflunix, Vet-Agro, Poland) 2.2 mg/kg b.w. i.v. once. At the beginning of treatment, all calves in the E groups received 100 mL of hyperimmune serum administered subcutaneously in two locations (50 mL each) on both sides of the thorax behind the shoulder.

The clinical evaluation and blood collection were performed for each calf included in the study: before treatment (SS1), on the 7th day (SS2), and 14th day after treatment (SS3). Blood was also collected from calves at 6 months of age (SS4). Blood from the jugular vein was taken to 2 mL tubes with K2EDTA (Profilab) and 11 mL with a clot accelerator and granule serum separator (Profilab). Body weight was measured on the 14th day after the beginning of treatment and at 6th month of age. Nasal swabs were collected before treatment initiation. Before sampling, nostrils were cleaned using sterile cotton wool tampons. Unguarded sterile swabs (Meus, Italy) were introduced 10–12 cm to the right nostril close to the nasal septum, rotated several times to the left and right, removed without contact with the external part of the nostril, placed on transport medium, and cultured under aerobic and microaerophilic conditions within 4 h of sampling. Blood and chocolate agar media (Graso, Poland) were used in this study. Microbiological analysis was performed by the Epi-Vet diagnostic laboratory (Wroclaw, Poland). The average daily weight gain in each calf was calculated as the difference in body weight between the 14th day after the beginning of the treatment and at 6th month of age, divided by the number of days for this period.

### Laboratory analysis

#### Presence of BoHV-1, BVDV, BRSV, PI-3V and *M. bovis* antibodies in serum obtained from hyperimmunized cows

In sera collected from hyperimmunized cows during the last three samplings after immunization (S5–S7), the presence of antibody against bovine herpesvirus-1 (BoHV-1), bovine viral diarrhea virus (BVDV), bovine respiratory syncytial virus (BRSV), bovine parainfluenza-3 virus (PI-3V), and *Mycoplasma bovis* were evaluated using an ELISA kit (Bio-X Diagnostics, BIO K 284). According to the test label, a sample was considered positive if it yielded a result greater than or equal to one plus sign (+ = 1). Frank seroconversion is considered to have occurred if the signal increases by two orders of magnitude (two pluses; e.g., +  +  >  +  +  +  + or +  >  +  + +). In cases where the results of the two evaluations increased by more than two (out of five), the result was considered seroconversion.

#### Detection of anti-*H. somni* rOMP40, anti-*H. somni *rHsp60 and anti-*A. pleuropneumoniae* LPS antibodies in serum obtained from hyperimmunized cows

Serum samples obtained from hyperimmunized cows (S1-S7 and pooled hyperimmune serum S5-S7) were examined for the presence of IgG_1,_ IgG_2_, IgM, and IgA antibodies against purified *H. somni* rHsp60, *H. somni* rOMP40, and *A. pleuropneumoniae* LPS. Indirect ELISA was performed as previously described, with some modifications^19,20^. Microplates (Nunc Maxisorp, Thermo Scientific) were coated with *H. somni* rHsp60 (3 µg/mL in 0.05 M carbonate buffer pH = 9.6, 100 µL per well), *H. somni* rOMP40 (3 µg/mL in phosphate-buffered saline/PBS pH = 7.4; 100 µL per well), or *A pleuropneumoniae* LPS (3 µg/mL in 0.05 M carbonate buffer, pH = 9.6 100 µL per well). The plates were coated for 2 h at 37 °C and incubated overnight at 4 °C. Than plates were washed three times using PBS buffer containing 0.05% Tween 20 (Merck KGaA, Germany; PBST; 200 μL per well). The plates were blocked with PBS buffer containing 1% Tween 20 (Merck KGaA; 200 μL per well; incubation for 90 min at 37 °C). To detect anti-*H. somni* rHsp60 antibody samples were diluted to 1:20 000 to determine the IgG_1_ and IgG_2_ subclass, 1:10 000 to determine the IgM class, and 1:100 to determine the IgA class. To detect anti-*H. somni* rOMP40 antibody samples were diluted: 1:4 000, 1:1000, 1:200, and 1:100 to determine the IgG_1,_ IgG_2_ subclass, and IgM and IgA class antibodies, respectively. For the detection of *A. pleuropneumoniae* LPS antibody samples were diluted to 1:500 for IgG_1_ and IgM classes and 1:100 for IgG_2_ and IgA classes. 100 μl of diluted serum samples were added to well, plates were incubated at room temperature (22 ± 2 °C) for 90 min. Plates were washed three times using PBST (200 μL per well). The dilutions of HRP-conjugated antibodies were 1:60 000 for sheep anti-bovine IgG_1_; 1:20 000 for sheep anti-bovine IgG_2_; 1:100 000 for rabbit anti-bovine IgM, and 1:10 000 for rabbit anti-bovine IgA (Bethyl Laboratories Inc., USA). 100 μL of conjugates were added to each wells. The plates were incubated at room temperature (22 ± 2 °C) in the dark for 90 min. Than plates were washed three times using PBST (200 μL per well). The color reaction was developed using a super-sensitive TMB substrate (100 μL per well; Merck) in the dark at room temperature for 15 min. The reaction was stopped by 2 M H2SO4 (50 μL per well; P.P.H. Stanlab Sp.J., Lublin, Poland). Absorbance was measured at a wavelength of 450 nm using an ELISA Microplate Reader μQuantum™ (BioTek Instruments, Vermont, USA). All samples were analyzed in duplicate. The absorbance data obtained were used for calculation.

#### Determination of immunoglobulin concentration in calves serum samples

The concentrations of immunoglobulins IgG_1_, IgG_2_ and IgM [g/L] in calf sera were determined using ELISA kits (Bovine IgG_1_/IgG_2_/IgM ELISA Quantitation Set Bethyl Laboratories Inc., USA). Different sample dilutions were used (1:40 000 for IgG_1_, 1:20 000 for IgG_2_ and 1:8 000 for IgM).

#### Acute phase protein concentration in calves serum samples; fibrinogen and hematocrit in calves’ blood

The serum concentrations of haptoglobin (Hp; g/L) and serum amyloid A (SAA; mg/L) were determined using ELISA kits (Cow Haptoglobin ELISA KIT Life Diagnostic, Inc. UK; Multispecies SAA ELISA kit; Tridelta Development Ltd., Ireland). Hematocrit [%] and fibrinogen [g/L] levels in whole blood were measured according to Millar et al.^27^.

#### Presence of BoHV-1, BVDV, BRSV, PI-3V and *M. bovis* antibodies in serum obtained from calves

Specific antibodies against BoHV-1, BRSV, BVDV, PI-3V, and *M. bovis* in the serum were evaluated (ELISA kit, Bio-X Diagnostics, BIO K 284) in SS1. The calves were not vaccinated until the end of the trail.

The group assignment of the animals was blinded to the laboratory technicians analyzing the samples.

### Statistical analysis

Statistical analyses were conducted using Statistica 12.5 (StatSoft Inc., Tulsa, OK, USA).

Data were analyzed for normality. In cows, anti-*H. somni* Hsp60 IgG_2_, and anti-*H. somni* OMP40 IgA and anti-*A.pleuropneumoniae* LPS IgM and IgA levels showed non-normal distributions. The logarithmic transformation of the data improved the normality of the anti-*H. somni* Hsp60 IgG_2_ and anti-*H. somni* OMP40 IgA. Except of anti-*A.pleuropneumoniae* LPS IgM and IgA class the reactivity of other antibodies in cows were analyzed by repeated-measures ANOVA with Duncan’s post hoc test at *p* ≤ 0.05, and *p* ≤ 0.01 levels. Data with non-normal distributions were compared using the Friedman ANOVA test with post hoc Wilcoxon comparison. Differences in weight gain between the end of treatment and 6th month of age in the examined groups of calves were compared using ANOVA. Ht, Fb, Hp, and SAA levels in calves showed a non-normal distribution; therefore, differences between groups in the corresponding samples were compared using Kruskal–Wallis ANOVA tests with multiple comparisons. Changes in Ht, Fb, Hp, and SAA in the repeated measures were tested using Friedman ANOVA, and post hoc comparisons between subsequent samplings were performed using the Wilcoxon test. The concentration of serum immunoglobulins in calves was tested using two-way factorial ANOVA, and post hoc comparisons were made using Tukey’s HSD test.

Clinical scores within groups were compared using Kruskal–Wallis ANOVA with multiple comparisons. The Pearson’s chi-square statistic for the distribution of sick and healthy animals between the groups during treatment (SS2 and SS3) was calculated, and post-hoc pairwise comparisons were performed using Fisher’s exact test with Bonferroni correction.

## Results

### Presence of BoHV-1, BVDV, BRSV, PI-3V and *M. bovis* antibodies in blood serum obtained from hyperimmunized cows

The occurrence and intensity of BoHV-1, BVDV, BRSV, PI-3V, and *M. bovis* antibodies in the serum of hyperimmunized cows are presented in Table 2.

**Table 2:** Humoral immune response against BoHV-1, BVDV, BRSV, PI-3V and *M. bovis* in the serum of cows at the time of blood collection for hyperimmune serum preparation.

Antibody against	Cow number	Time of blood collection for serum		
		S5	S6	S7
BoHV-1	I	4	2	2
	II	5	4	4
	III	5	4	5
	IV	5	4	5
	V	4	4	5
BVDV	I	**2**	**4**	3
	II	**1**	**3**	2
	III	1	2	1
	IV	**1**	**3**	1
	V	4	**2**	**4**
BRSV	I	3	4	4
	II	2	3	2
	III	3	3	4
	IV	2	3	2
	V	4	4	5
PI-3V	I	3	3	3
	II	2	2	2
	III	3	3	3
	IV	3	3	4
	V	**1**	**3**	2
*M. bovis*	I	0	0	0
	II	0	0	0
	III	0	0	0
	IV	0	0	0
	V	0	0	0

I-V: hyperimmunized cow number; S5-S6-S7: sampling. Sera were obtained as follows: S5: immediately before the fifth immunization; S6: immediately before the sixth immunization; S7: three weeks after the sixth immunization; BoHV-1: bovine herpesvirus-1; BVDV: bovine viral diarrhea virus; BRSV: bovine respiratory syncytial virus; PI-3V: bovine parainfluenza-3 *virus*; M. bovis: *Mycoplasma bovis*. Seroconversion of specific antibodies is bolded.

All five hyperimmunized cows were positive for BoHV-1, BVDV, BRSV, and PI-3V antibodies and negative for *M. bovis* antibody (Table 2). During hyperimmunization, a significant increase in antibody reactivity was detected for BVDV in four cows and for PI-3V in one animal.

### Detection of anti-*H. somni* rOMP40 and anti-*H. somni* rHsp60 antibodies in blood serum obtained from hyperimmunized cows

A significant increase (*p* ≤ 0.01) in the serum reactivity of IgG_1_ antibody against *H. somni* rHsp60 and *H. somni* OMP40 antigens was detected two weeks after the 2nd immunization (S3; Table 3), and a significant difference (*p* ≤ 0.01) with pre-hyperimmunization (the first sampling; S1) was observed until the last sampling. In contrast to IgG_1_, the intensity of the serum IgG_2_ antibody reaction against *H. somni* rHsp60 and *H. somni* rOMP40 antigens increased significantly faster—two weeks after the first immunization (S2; *p* ≤ 0.05), and a significant difference (*p* ≤ 0.01) from pre-hyperimmunization (S1) was observed until the last sampling. A significant increase in IgM antibody reactivity against *H. somni* rHsp60 was noted two weeks after the third immunization (S4; *p* ≤ 0.05), and in S5-S7 the intensity was significantly higher (*p* ≤ 0.01) than that in S1. A significant increase (*p* ≤ 0.05) in the IgM antibody against *H. somni* OMP40 was noted two weeks after the 2nd immunization (S3), and the intensity was higher than that before hyperimmunization until the end of trial (*p* ≤ 0.01). In the IgA class, the immune response was found only against *H. somni* rHsp60, and significantly higher reactivity occurred between the 5th and 7th sampling (*p* ≤ 0.05, for S5 and S6; *p* ≤ 0.01 for S7). Five repeated injections of the antigenic preparation were necessary to significantly increase the anti- *A. pleuropneumoniae* LPS reactivity in the IgG_1_ class (*p* ≤ 0.05) and six in the IgG_2_ class (*p* ≤ 0.01). No significant changes in antibody reactivity were detected for *A. pleuropneumoniae* LPS in the IgM and IgA classes, or against *H. somni* OMP40 in the IgA class. The humoral immune response of cows against *A. pleuropneumoniae* LPS in the IgM class was heterogenous at S5 and S7, as indicated by the SD value.

**Table 3:** The mean intensity of serum antibody reactions ± SD in ELISA (absorbance 450 nm) in five hyperimmunized cows against *H. somni* rHsp60 and rOMP40 and *A. pleuropneumonie* whole LPS.

Solid phase antigen used in ELISA	Ab class	Sampling time							Pooled serum
		S1	S2	S3	S4	S5	S6	S7	PS5-7
*H. somni* rHsp60	IgG_1_	0.130^A^ ± 0.02	0.463 ± 0.24	0.793^B^ ± 0.10	1.713^B^ ± 0.17	1.960^B^ ± 0.55	1.554^B^ ± 0.33	1.325^B^ ± 0.47	1.817 ± 0.04
	IgG_2_	0.099^Aa^ ± 0.01	0.191^b^ ± 0.08	0.349^B^ ± 0.15	0.676^B^ ± 0.25	1.158^B^ ± 0.64	1.095^B^ ± 0.60	1.289^B^ ± 0.70	1.440 ± 0.04
	IgM	0.249^Aa^ ± 0.07	0.304 ± 0.05	0.280 ± 0.09	0.402^b^ ± 0.14	0.532^B^ ± 0.22	0.505^B^ ± 0.18	0.687^B^ ± 0.18	0.677 ± 0.02
	IgA	0.429^Aa^ ± 0.15	0.473 ± 0.13	0.473 ± 0.15	0.630 ± 0.33	0.691^b^ ± 0.24	0.742^b^ ± 0.19	0.777^B^ ± 0.34	0.802 ± 0.01
*H. somni* rOMP40	IgG_1_	0.112^A^ ± 0.02	0.552 ± 0.55	0.784^B^ ± 0.21	1.090^B^ ± 0.39	1.041^B^ ± 0.47	0.774^B^ ± 0.38	0.619^B^ ± 0.40	0.829 ± 0.02
	IgG_2_	0.139^A^ ± 0.02	0.625^B^ ± 0.53	1.096^B^ ± 0.55	1.543^B^ ± 0.48	1.708^B^ ± 0.45	1.442^B^ ± 0.45	1.138^B^ ± 0.27	1.336 ± 0.02
	IgM	0.571^Aa^ ± 0.19	0.683 ± 0.17	0.824^b^ ± 0.16	1.016^B^ ± 0.23	1.009^B^ ± 0.20	0.857^b^ ± 0.13	0.982^B^ ± 0.28	0.853 ± 0.02
	IgA	0.116 ± 0.03	0.099 ± 0.02	0.105 ± 0.03	0.127 ± 0.06	0.133 ± 0.04	0.133 ± 0.03	0.116 ± 0.03	0.139 ± 0.02
*A. pleuropneumoniae* LPS	IgG_1_	0.745^a^ ± 0.27	0.722 ± 0.25	0.719 ± 0.24	0.883 ± 0.20	0.817 ± 0.20	0.930^b^ ± 0.20	0.897^b^ ± 0.33	0.987 ± 0.14
	IgG_2_	0.175^A^ ± 0.04	0.168 ± 0.05	0.187 ± 0.05	0.198 ± 0.04	0.217 ± 0.04	0.204 ± 0.03	0.246^B^ ± 0.05	0.276 ± 0.04
	IgM	0.364 ± 0.09	0.397 ± 0.10	0.399 ± 0.09	0.405 ± 0.07	0.403 ± 0.14	0.335 ± 0.02	0.497 ± 0.18	0.395 ± 0.06
	IgA	0.120 ± 0.03	0.110 ± 0.02	0.115 ± 0.03	0.117 ± 0.03	0.118 ± 0.03	0.117 ± 0.02	0.139 ± 0.05	0.150 ± 0.01

S1-S7: samples taken during immunization; P: pooled serum collected at S5, S6, and S7 used in the treatment of calves (due to one sample not included in the analysis). Values with different small letters (ab) differ between sampling at *p* ≤ 0.05. Values with different capital letters (AB) differ between sampling at *p* ≤ 0.01.

### Bacteria isolated from calves

In cases of three calves in the CM group, five calves in the EM group, and one calf in the ES group, the swabs cultures indicate a positive result, and *Pasteurella* ssp. were identified. *P. multocida* was isolated in swab culture from one calf in the CM and CS groups and in three calves in the ES group. No *H. somni* or *M. hemolytica* were isolated.

### Calves health score

The highest clinical score was observed at the beginning of the treatment (SS1) (Table 4). During treatment, the score significantly decreased in SS2 (*p* ≤ 0.05 in EM group and *p* ≤ 0.01 in the remaining groups) and SS3 (*p* ≤ 0.05, CM, and *p* ≤ 0.01, remaining groups) time points compared to SS1 in all treatment groups.

**Table 4:** Median (mean ± SD) clinical score and percentage of calves with clinical score 0 during treatment of respiratory disease in treatment groups.

Group	Sampling		
	SS1	SS2	SS3
EM (n = 10)	6.0 (5.6 ± 0.5)^aA^ 0%	2.0 (2.3 ± 1.9)^b^ 20%	0.0 (0.2 ± 0.6)^B^ 90%
ES (n = 10)	13.0 (12.9 ± 1.5)^A^ 0%	3.5 (3.4 ± 1.9)^B^ 10%	2.0 (1.5 ± 1.4)^B^ 40%
CM (n = 10)	6.5 (6.7 ± 1.8)^Aa^ 0%	2.0 (2.7 ± 1.7)^B^ 10%	4.0 (3.5 ± 2.8)^b^ 30%
CS (n = 10)	11.5 (12.1 ± 1.3)^A^ 0%	3.0 (4.6 ± 3.0)^B^ 0%	3.0 (3.6 ± 2.4)^B^ 10%

EM: group treated with serum with moderate signs of respiratory disease; ES: group treated with serum with severe signs of respiratory disease; CM: group treated without serum with moderate signs of respiratory disease; CS: group treated without serum with severe signs of respiratory disease; SS1-SS3: following sampling. Values with different lowercase letters (ab) differ within groups between samples, *p* ≤ 0.05. Values with different capital letters (AB) differ between the groups (*p* ≤ 0.01).

In the first sampling, none of the calves scored 0 points; in the second sampling, two calves in the EM group and one calf in the ES and CM groups scored 0 points. In the last sampling, nine calves in the EM, four calves in the ES, three calves in the CM, and one calf in the CS showed no clinical signs of respiratory disease (received 0 points). The proportions of sick and healthy calves between the groups in SS3 were significantly different (P(χ2) ≤ 0.01). Post hoc comparisons revealed that significantly more calves were healthy at SS3 in the EM group compared to the CS group (*p* ≤ 0.01). None of the calves died up to 6 months of age.

### Presence of BoHV-1, BVDV, BRSV, PI-3V and *M. bovis* antibodies in blood serum obtained from calves

The majority of calves had serum antibodies against BoHV-1, BVDV, BRSV, and PI-3V on the first day of BRD treatment, and a supplementary file shows this in detail (see Supplementary file Table S2). The antibody against *M. bovis* was detected in one calf in the EM, ES, and CM groups and in three calves in the CS group.

### Calves body weight gain

The age of the calves in the treatment groups and their weight gain are included in a supplementary file Table S3 (see Supplementary file 3 Table S3).

The ages of the calves at the beginning of the study were not significantly different between the groups. There was high individual variation in the mean weight gain in each group (Table S3). The average daily gain did not differ significantly between the treatment groups.

### Missing samples

In the determination of IgG_1_ concentration, in the EM group, one sample was missing from SS3 (n = 9), and in the ES group, one sample was missing from SS1 and SS3 (n = 9), and three samples were missing from SS4 (n = 7). In the determination of IgG_2_ concentration in the ES group, one sample was missing from SS1, SS3, and SS4 (n = 9). In the determination of the IgM concentration, in the ES group, one sample was missing from SS1, SS3 (n = 9) and two from SS4 (n = 8). In the determination of SAA, one sample was missing from the SS4 in the ES group (n = 9).

### Acute phase protein concentration in calves serum samples and hematocrit in calves’ blood

The hematocrit levels and concentrations of fibrinogen, haptoglobin, and serum amyloid A in calves are shown in Table 5. In the CS group, the HCT levels were significantly higher in SS1 (*p* ≤ 0.05) and SS3 (*p* ≤ 0.01) than in SS4. Significantly higher HCT levels were observed at the last sampling in the EM and CM groups than in the CS group (*p* ≤ 0.05). However, the mean HCT value was within the reference interval calculated for the Holstein Friesian calves^28^. Higher concentrations of Fb were observed at SS1. In the CM and CS groups, the Fb concentration at SS1 was significantly higher than that at SS3 and SS4 (*p* ≤ 0.05). The concentration of Hp in SS1 in the ES group was significantly higher than that in SS2, SS3, and SS4 (*p* ≤ 0.05). In the CS group, such a difference was observed only between SS1 and SS2 (*p* ≤ 0.05). In SS1, the highest SAA concentration was observed in the ES group, which was significantly higher than that in the CM group (*p* ≤ 0.01). In the EM group, SAA concentrations in SS1 were significantly higher than those in SS2, SS3 (*p* ≤ 0.05), and SS4 (*p* ≤ 0.01). In the ES group, SAA concentrations in SS1 were significantly higher than those in SS2, SS3 (*p* ≤ 0.01), and SS4 (*p* ≤ 0.05).

**Table 5:** Median (mean ± SD) concentrations of hematocrit, fibrinogen, haptoglobin and serum amyloid A in the treatment groups.

Parameter	Sampling	EM	ES	CM	CS
		n = 10	n = 10	n = 10	n = 10
HCT (%)	SS1	35.0 (33.7 ± 4.9)	36.0 (35.6 ± 2.4)	36.0 (35.5 ± 2.6)	35.0^a^ (34.1 ± 2.6)
	SS2	35.5 (34.6 ± 5.0)	34.5 (34.9 ± 3.6)	35.5 (35.9 ± 3.0)	33.0 (29.5 ± 10.6)
	SS3	35.3 (34.6 ± 4.4)	35.0 (35.4 ± 2.2)	36.0 (36.5 ± 2.3)	35.0^A^ (34.7 ± 2.5)
	SS4	35.0^2)^ (34.0 ± 2.7)	33.0 (33.0 ± 1.8)	35.0^2)^ (34.2 ± 3.3)	30.5^1)Bb^ (30.4 ± 1.6)
Fb (g/L)	SS1	6.2 (6.5 ± 2.1)	5.6 (5.9 ± 1.6)	6.8^a^ (6.0 ± 2.3)	5.7^a^ (6.2 ± 1.6)
	SS2	5.0 (5.1 ± 2.1)	4.9 (5.2 ± 1.6)	5.1 (5.1 ± 0.5)	5.2 (5.1 ± 0.7)
	SS3	5.3 (5.1 ± 0.9)	4.9 (4.9 ± 1.0)	4.0^b^ (4.1 ± 1.3)	5.6 (5.3 ± 1.2)
	SS4	5.0 (5.1 ± 1.7)	5.1 (5.0 ± 0.8)	5.2 (5.4 ± 1.5)	4.3^b^ (4.1 ± 1.7)
Hp [g/L]	SS1	0.0 (0.1 ± 0.2)	0.1^a^ (0.1 ± 0.1)	0.0 (0.0 ± 0.0)	0.0^a^ (0.1 ± 0.2)
	SS2	0.0 (0.0 ± 0.0)	0.0^b^ (0.0 ± 0.0)	0.0 (0.0 ± 0.0)	0.0^b^ (0.0 ± 0.0)
	SS3	0.0 (0.04 ± 0.1)	0.0^b^ (0.0 ± 0.0)	0.0 (0.0 ± 0.0)	0.0 (0.0 ± 0.1)
	SS4	0.0 (0.0 ± 0.0)	0.0^b^ (0.0 ± 0.0)	0.0 (0.1 ± 0.2)	0.0 (0.0 ± 0.0)
SAA (mg/L)	SS1	74.8^Aa^ (80.1 ± 62.3)	104.1^4)Aa^ (129.7 ± 77.9)	18.3^3)^ (30.1 ± 37.6)	73.8 (77.7 ± 66.3)
	SS2	5.0^b^ (28.5 ± 45.1)	27.5^B^ (46.1 ± 47.4)	10.0 (17.9 ± 23.0)	24.0 (30.9 ± 29.7)
	SS3	28.2^b^ (41.0 ± 40.3)	42.5^B^ (54.4 ± 36.1)	30.3 (35.3 ± 33.9)	20.6 (45.3 ± 55.8)
	SS4	0.0^B^ (17.4 ± 44.2)	47.8^b^ (45.6 ± 40.5) ^(n=9)^	3.5 (28.3 ± 48.2)	19.9 (39.3 ± 54.3)

EM: group treated with serum with moderate signs of respiratory disease; ES: group treated with serum with severe signs of respiratory disease; CM: group treated without serum with moderate signs of respiratory disease; CS: group treated without serum with severe signs of respiratory disease; HTC: hematocrit, Fb: fibrinogen, Hp: haptoglobin, and SAA: serum amyloid A; SS1-SS4: following sampling. Values with numbers 1) and 2) differ between groups at *p* ≤ 0.05 groups in the same sampling. Values with numbers 3) and 4) differed (*p* ≤ 0.01 between groups in the same sampling). Values with different lowercase letters (ab) differ within groups between samples, *p* ≤ 0.05. Values with different capital letters (AB) differ within groups between samplings, *p* ≤ 0.01.

### Immunoglobulin concentration in calves serum

There were no differences between the groups or within the sampling of IgG_1_ immunoglobulin concentration in calf serum. IgG_2_ at the age of 6 months was numerically the highest in the ES group, which showed severe signs of respiratory infection at the start of treatment (Table 6). Additionally, the ES group in SS4 had the highest IgM concentration, which was significantly different from that of the CS group (*p* ≤ 0.05).

**Table 6:** Mean ± SD IgG_1_, IgG_2_ and IgM concentrations in the treatment groups.

Ig class	Sampling	EM	ES	CM	CS
		n = 10	n = 10	n = 10	n = 10
G_1_	SS1	15.3 ± 12.8	13.4 ± 3.0 ^(n=9)^	9.3 ± 6.1	11.4 ± 4.3
	SS2	12.0 ± 4.2	12.1 ± 5.1	10.4 ± 5.2	9.9 ± 3.6
	SS3	12.0 ± 3.8 ^(n=9)^	14.4 ± 5.9 ^(n=9)^	9.6 ± 1.9	8.9 ± 3.4
	SS4	13.1 ± 3.2	15.1 ± 4.2 ^(n=7)^	11.3 ± 3.2	9.1 ± 2.1
G_2_	SS1	3.5 ± 1.8	5.8 ± 3.3 ^(n=9)^	4.2 ± 2.2	6.2 ± 3.0
	SS2	3.6 ± 1.3	6.2 ± 3.0	4.9 ± 2.8	5.0 ± 3.2
	SS3	5.0 ± 3.6	6.7 ± 3.2 ^(n=9)^	3.8 ± 1.5	5.6 ± 2.8
	SS4	7.3 ± 3.1	11.3 ± 3.4 ^(n=9)^	6.9 ± 3.0	7.7 ± 1.7
M	SS1	1.1 ± 0.6	1.3 ± 0.6 ^(n=9)^	0.9 ± 0.4	1.7 ± 1.2
	SS2	0.9 ± 0.5	1.9 ± 1.6	0.9 ± 0.4	1.2 ± 0.5
	SS3	1.1 ± 0.4	1.8 ± 1.3 ^(n=9)^	1.1 ± 0.4	1.3 ± 0.8
	SS4	1.9 ± 0.6	3.5 ± 2.3 ^(n=8) a^	2.0 ± 0.9	1.7 ± 0.5^b^

EM: group treated with serum with moderate signs of respiratory disease; ES: group treated with serum with severe signs of respiratory disease; CM: group treated without serum with moderate signs of respiratory disease; CS: group treated without serum with severe signs of respiratory disease; SS1-SS4: following sampling; Ig: immunoglobulin. Values with different capital letters (ab) differ at *p* ≤ 0.05.

## Discussion

The aim of this study was to produce bovine hyperimmune serum from cows immunized with a formulation containing selected, conserved proteins from *H. somni* (rHsp60 and rOMP40) and their conjugates with the LPS core oligosaccharide of *A. pleuropneumoniae*. The effect of supplementing calves with this antiserum was then evaluated in the context of respiratory disease treatment.

The application of hyperimmune serum for the treatment of viral or bacterial diseases in calves is not a new idea^15,29^. However, methods used to increase the specificity of antibodies in sera have changed over the years^14,29,30^. In recent years, a commercially available vaccines^14^ and recombinant proteins^25,30^ have been used to produce the hyperimmune bovine serum/plasma. Our previous study^21^ showed that calves respond very fast in IgG_1_ and IgG_2_ to *H. somni* rHsp60 immunization. In that study^21^, after two s.c. immunizations, there was no response in the IgM class; in the present study, cows showed increased serum IgM reactivity after the third immunization. A significant increase in the IgA class took even longer. This was surprising because IgM antibodies are produced in the primary immune response^31^. We hypothesized that the increase in IgM antibody reactivity after the third immunization was a result of rHsp60- LPS conjugate recognitions. LPS is an anamnestic antigen that is recognized by B cells in a T-cell-independent manner and induces a specific immune response in the IgM class of antibodies^32^. Most likely, the high initial reactions in different classes suggest some level of recognition and earlier contact of cows with this protein and/or bacteria. This is not surprising, since Hsp60 is known to be highly conserved and widely spread in nature, with highly conserved proteins found in all prokaryotic and eukaryotic cells^33^. Particularly in bacteria, the expression of Hsp60 on the surface occurs constitutively and increases remarkably during host infection^34^. Natural exposure of cattle to environmental Gram-negative bacteria, especially those that are present in the digestive tract, may induce a humoral immune response with cross-reactivity of antibodies against other pathogenic Gram-negative bacteria^35^.

*Histophilus somni* OMP40 is a major outer membrane protein^36^. During illness, some outer membrane proteins are recognized and the humoral immune response develops. Convalescent sera from calves experimentally infected with *H. somni* strongly reacted with the outer membrane proteins (78 and 40 kDa) of this bacterium, as shown by western blotting^37^. Sera obtained from vaccinated cattle, swine, dogs, horses, and poultry with whole *H. somni* cells showed a strong immune response against selected *H. somni* antigens, among others, against major outer membrane proteins^18^. In our study, repeated vaccination with recombinant OMP40 from *H. somni* resulted in a significant humoral response. The pattern of cow response to immunization with this protein was similar to that of *H. somni* rHsp60 in the IgG_1_, IgG_2_ and IgM classes. In a previous study^25^, beef cows hyperimmunized with *H. somni* rHsp60 and rOMP40 showed a significant increase in IgG_1_ and IgG_2_ antibodies as early as 2 weeks after the first immunization and maintained at an elevated level until the end of observation (3 weeks after the 6th dose), but no significant increase in antibodies was observed in the IgM class. This suggests that LPS is required for IgM response in the presence of preliminary recognition.

A similar composition of antigens to those used in the present study was used to produce hyperimmune serum in porkers^24^. The IgG immune response of porkers against the examined proteins was similar, but the reactivity of IgM anti-*H. somni* rHsp60 and anti-*H. somni* rOMP40 antibodies after the initial significant increase (after the second immunization) decreased remarkably, which was not observed in this study. It appears that in cattle, hyperimmunization allows for a continuous increase in IgM antibody levels. Surprisingly, a significant increase in LPS *A. pleuropneumoniae* antibody reactivity in hyperimmunized cows occurred after 5th immunization in IgG_1_ and after 6th dosis in the IgG_2_ class, but in contrast to a study in porkers^24^, no significant rise in IgM antibody was found. Elevated antibody recognition of lipopolysaccharides (LPS), particularly from pathogens such as *S.* Dublin, has been observed in heifers and cows from diverse herds. Animals exposed to specific strains develop substantial antibody responses, primarily because of repeated exposure or persistent infections^38^. Because of the initial high recognition, multiple vaccinations were necessary to elicit an increased specific response. In one study^35^, steers immunized with the commercial vaccine J5 *E. coli* showed significantly increased serum IgM and IgG_1_ reactivity after two doses of the vaccine, and this increased reactivity was maintained until the end of the immunization schedule (12 times), but it took longer to induce an IgG_2_ response (5 doses). The different patterns of immunoglobulin responses in isotypes and classes (Th1 and Th2 responses) in cattle are a phenomenon that may arise from different factors, such as antigen dose, type and affinity to T-cell receptors, and the cytokine environment during antigen priming, which can be influenced by the nature of the pathogen or antigen^39^. Therefore, to produce serum with strong antigen-specific recognition and cross-reactivity with other bacteria, multiple doses are recommended. To induce increased titers and cross-reactivity to heterologous Gram-negative bacteria, three or more immunizations are necessary^35^. In our study, six doses were sufficient to induce a response in the majority of the examined classes against the proteins used, and this is the same number of immunizations suggested in the study by Chaiyotwittayakun et al.^35^.

In addition to evaluating the immunization effect, the presence of antibodies against the most common pathogens was determined. Antibodies against BoHV-1, BVDV, BRSV, and PI-3V were present in cows that were used as donors. As in herds, vaccination against BoHV-1 and BVD viruses was performed in adult animals, and the presence of these antibodies was expected. The BRSV and PI-3V antibodies, since no vaccination against these viruses was performed in the herd due to contact of cattle with those viruses, are often involved in BRD outbreaks in cattle^40^. Because the hyperimmune serum utilized in this study contained both specific antibodies against bacterial antigens and viruses of respiratory disease, it is difficult to evaluate the role played by both types of antibodies during treatment.

The risk of respiratory diseases in dairy heifers is high during the first few months of life^1^. The youngest calf included in the study was 19 days old and the oldest was 98 days old. This large age variation is a limitation of the study and obscures the interpretation of the presence of specific antibodies against the examined viruses and *M. bovis* in the serum. In most cases, it could be assumed that these antibodies were probably derived from colostrum. The time necessary to disappear the colostrum derived antibody is more than 6 months for BVDV, five months for BRSV and PI-3V and almost four months for BoHV-1^41^. We do not know the levels of passive transfer from calves, so it is impossible to draw conclusions about the presence of antibodies against viruses; however, since herd vaccination against BVDV and BoHV-1 was present, the high prevalence of these antibodies was expected. The low incidence of calves with serum antibody against *M. bovis* may reflect a low exposure rate to this agent in the herd^42^, as cows were not vaccinated and no cow donors with hyperimmune serum showed the anti-*M. bovis* antibody.

Key bacterial pathogens associated with bovine pneumonia include *P. multocida*, *M. hemolytica*, *H. somni*, *M. bovis*, and other *Mycoplasma* spp.^43–45^. The most common respiratory tract pathogen isolated in this study from dairy calves with BRD was *Pasteurella* which is commonly detected in calves^43,46^. Nevertheless, a limitation of the swab method used in this study exists. Results obtained from unguarded nasal swabs demonstrated a higher prevalence of polymicrobial growth than guarded swabs^47^. An additional limitation is that the isolated bacteria may be present in the nasal cavity of healthy calves; however, a moderate agreement exists between the results collected from deep nasal swabs and bronchoalveolar lavage^5^ so it may be assumed that the isolated bacteria may have been involved in the pathogenesis of BRD in these calves. Lack of *H. somni* isolation may be concerning, since this pathogen is one of the causes of BRD, however the method of sample collection can significantly affect the likelihood of isolating *H. somni*. It has been shown that *H. somni* is more likely to be isolated from bronchoalveolar lavage (BAL) samples than from deep nasopharyngeal swabs^48^.

Average daily weight gain (ADG) was not influenced by serum or disease severity, but there was high variation within groups. In the current study, the ADG was slightly lower than that in the study by Overton^1^, where for heifers with BRD between 2 and 4 months of age and 4 and 10 months of age, the ADGs were 0.92 and 0.89 kg per day, respectively.

A significant decrease in the clinical score during the treatment showed that calves were treated successfully; however, the highest number of healthy animals was in the group that had moderate signs of respiratory diseases, and serum was used in addition to the standard treatment protocol on the farm. The lower effectiveness of the treatment without the additive use of hyperimmune serum, which was seen in the controls with severe level of inflammation, might be associated with incomplete resolution of the initial lung infection^49^. In previous study^50^ subcutaneous application of hyperimmune serum prepared by multiple revaccinations using different routes (i.m., s.c., i.v. i.n) of the donor immunized with the killed and live reference strain J282 *M. bovis* to calves that were infected intranasally with *M. bovis* did not succeed in decreasing clinical manifestations or pathological alterations in the lungs of experimentally infected calves, but this may be dependent on the dose to the exposed pathogen. Specific antibodies against *H. somni* rHsp60 inhibit the production of *H. somni* biofilms in vitro^51^. The ability to produce biofilms in respiratory pathways and other organs plays an important role in *H. somni* colonization and the survival of mucosal membranes^52^. It can be expected that the antibodies present in hyperimmune serum may disrupt the metabolic activity of Gram-negative pathogens responsible for respiratory tract infections, as the intramuscularly administered antibody in dogs was secreted into the respiratory tract^53^. In calves, not only sIgA but also IgG_1_ antibodies are present in the upper respiratory tract secretions^54^. However, this hypothesis requires further confirmation in future studies. Interesting results of intratracheal application of hyperimmune serum were obtained by Haralambiev et al.^15^. They suggested that intratracheal inoculation with antibodies may imitate the natural defense of the respiratory mucosa against viruses. The same authors compared, in a small group of calves, the effects of intratracheal (3–5 mL; n = 51) and intramuscular (20 mL; n = 59) administration of the same hyperimmune serum. Approximately 8% of the intratracheally treated calves became ill, compared to 20% in the intramuscular group. This observation suggests that the direct application of antibodies on the mucosal membrane of respiratory pathways should be evaluated as possibly more efficient than subcutaneous/intramuscular in future studies.

The anti-core LPS antibody was expected to protect calves in this study against the harmful effects of LPS, including sepsis^22,55,56^. The cross-reactivity of anti-core LPS antibodies has been exploited in the treatment of rat sepsis^55^. As none of the calves in our study died, no cases of sepsis were observed.

We cannot exclude the possibility that the combination of serum and antibiotics had a higher success rate in decreasing the clinical score and number of calves that were healthy at the third sampling. In a mouse pneumonia model caused by *Acinetobacter baumanii*^57^, the combination of serum developed against outer membrane vesicles (AbOMVs) and levofloxacin improved levofloxacin sensitivity, which significantly reduced the bacterial loads in the lung and spleen compared to those of the antibiotic or antibody alone. The combination (antibiotics and antibodies) also significantly reduced lung inflammatory cell infiltration and cytokine aggregation^57^. It should be considered, that antibiotics may have varying effects on immune cells. Tetracyclines, such as oxytetracycline, have been shown to suppress the phagocytic capacity of macrophages^58^, whereas fluoroquinolones^59^ and cephalosporins^60^ have been found to enhance macrophage phagocytosis and intracellular bacterial killing. Therefore, the final effect may depend on the type of antibiotic used.

In calves, age-dependent changes in blood biochemical and hematological profiles are noted, and during the first weeks of life, the values of HCT are usually higher than those in the following weeks^61^. The value of HCT was similar to that presented in other studies^61,62^, and in the last sampling, the variability between calves was the lowest, as reported by Brun-Hansen et al.^62^ as age related. However, calves that experienced severe BRD exhibited significantly lower HCT levels at six months post-treatment. This finding, coupled with the numerically lowest mean body weight gain, suggests that BRD, or potentially other illnesses, influence calves development.

Bacterial, viral, and mixed respiratory tract infections usually cause systemic acute phase responses reflected by an increase in acute phase proteins (APPs)^63–65^. In this study, three acute-phase proteins (Fb, Hp, and SAA) were measured to assess the severity of respiratory inflammation and monitor the effects of treatment. The final sampling, at six months of age, aimed to confirm the final health status of the animals. At the initial sampling, the very low mean concentration of Hp showed that the production of this protein did not occur in all animals, which is common. Similarly, in a previous study^66^, low Hp levels (< 25 mg/L) were observed in over 66% of five-month-old beef calves with BRD, suggesting that Hp is not a highly sensitive marker for detecting BRD. In this studies, a decline in Hp concentration following treatment indicated a resolution of the acute inflammatory process, regardless of whether hyperimmune serum was used. Treatment also led to a decrease in Fb concentration, although a significant reduction within two weeks was only seen in the CM group. The threshold for SAA in healthy calves was calculated to be 25.6 mg/L^67^, and in our study, the majority of calves had SAA values above this level at the beginning of treatment. Because groups were divided based on the severity of clinical signs, which in cattle correlates with APP concentrations^68^, initial values reflected the clinical condition of the calves. However, a significant decrease in SAA during treatment was observed only in the groups that received serum supplementation. In the last sampling at 6th month of age, some calves exhibited elevated SAA concentrations without corresponding rise in Hp. This finding supports that SAA is a more sensitive protein in detecting the inflammatory process than Hp^69^.

Calves protected with good-quality colostrum reached the lowest IgG concentration at approximately 4–6 weeks of age, and then in the following weeks, IgG gradually increased again^70^, therefore, the majority of detected fluctuations of IgG_1_ and IgG_2_ in subsequent samplings may be influenced by age, not by the diseases themselves. In this study, at the last sampling, the ES group had a significantly higher concentration of IgM as well as an increased IgG_2_ concentration than calves in the CS group. IgM antibodies in cattle are important for the primary immune response and act as agglutinating antibodies in the serum^71^. Both IgM and IgG_2_ have opsonic and complement-binding activity in cattle^72–74^. Bovine IgG_2_ antibodies play a critical role in protection against pyogenic infections^72,75,76^; thus, an increase in this class of immunoglobulins may suggest early-stage infection with such pathogens in these calves, which is consistent with the increase in SAA. This may also indicate that the passively acquired antibodies, which improved the treatment effectiveness after hyperimmune serum injection on the first day of treatment, were not present at this time. Since calf-hood respiratory disease in heifers is associated with negative long-term outcomes, including increased mortality, reduced ADG, delayed age at first calving, diminished future milk production^4^; the incidence of BRD experienced by these calves might also increase the risk of subsequent diseases.

## Conclusion

Hyperimmunization of cows using selected antigens allowed the production of hyperimmune serum containing high levels of IgG_1_ and IgG_2_ antibodies against *H. somni* rHsp60, *H. somni* rOMP40, and *A. pleuropneumoniae* lipopolysaccharides. The subcutaneous application of hyperimmune serum, as a supplement to antibiotic and NSAID treatment of respiratory tract infections in dairy heifer calves, resulted in a significantly higher number of clinically healthy calves at the final sampling in the EM group compared to the CS group.

## Electronic supplementary material

Below is the link to the electronic supplementary material.

Supplementary Material 1

## Data Availability

The datasets generated and/or analysed during the current study are available in the “Hyperimmune serum containing *Histophilus somni* rHsp60, *H. somni* rOMP40 and *Actinobacillus pleuropneumoniae* LPS core oligosaccharide antibodies as supplementary treatment of respiratory tract diseases in dairy heifer calves.” repository, DOI: 10.57755/gzc8-9258, https://bazawiedzy.upwr.edu.pl/info/researchdata/UPWRf44da333910c4e88ad45482def5fcb23/.

## Abbreviations

BRD: Bovine respiratory disease
CM: Control group of claves with moderate clinical signs
CS: Control group of claves with severe clinical signs
EM: Experimental group of claves with moderate clinical signs
ES: Experimental group of claves with severe clinical signs
rOMP40 * H. somni*: Recombinant outer membrane protein 40 kDa obtained from *Histophilus somni*
rHsp60 *H. somni*: Recombinant heat shock protein 60 kDa obtained from *Histophilus somni*
S1-S7: Sampling obtained from hyperimmunized cows
SS1-SS4: Sampling obtained from calves
s.c.: Subcutaneous immunization
