# Supplementary material for: Hyperimmune serum containing Histophilus somni rHsp60, rOMP40 and Actinobacillus pleuropneumoniae LPS antibodies as supplementary treatment for calf respiratory diseases
Source: Sci Rep. 2025 May 30;15:19088. doi: 10.1038/s41598-025-03624-1 (PMC12125373; doi:10.1038/s41598-025-03624-1)
Supplement: Supplementary file 1 — Supplementary Material 1 [file 41598_2025_3624_MOESM1_ESM.docx]

**Supplementary file 1. Table S1. Composition of total mix ration (TMR)**

| **Component** | **DM %** | **kg** | **Percentage in TMR** |
| --- | --- | --- | --- |
| Soybean meal | 88 | 0,25 | 2,3 |
| Barley grain | 88 | 1,5 | 13,82 |
| Rindavit start | 98 | 0,1 | 0,92 |
| Corn silage | 34,5 | 4,0 | 36,87 |
| Alfaalfa silage | 27,1 | 4,0 | 36,87 |
| Grass silage | 38,9 | 1,0 | 9,22 |

Legend: DM: dry matter

**Supplementary file 2. Table S2. The presence (%) of antibody against- BoHV-1, BVDV, BRSV, PI-3V and *Mycoplasma bovis* in the serum of calves at the first day of treatment.**

| **Group** | **Presence of antibody [%]** | | | | |
| --- | --- | --- | --- | --- | --- |
|  | **BoHV-1** | **BVDV** | **BRSV** | **PI-3V** | ***M. bovis*** |
| EM n=10 | 90 | 70 | 80 | 80 | 10 |
| ES n=10 | 90 | 80 | 70 | 90 | 10 |
| CM n=10 | 90 | 40 | 50 | 70 | 10 |
| CS n=10 | 70 | 60 | 70 | 90 | 30 |

Legend: EM: group treated with serum with moderate signs of respiratory disease; ES: group treated with serum with severe signs of respiratory disease; CM: group treated without serum with moderate signs of respiratory disease; CS: group treated without serum with severe signs of respiratory disease; BoHV-1: Bovine Herpesvirus-1; BVDV: Bovine Viral Diarrhea Virus; BRSV: Bovine Respiratory Syncytial Virus; PI-3V: Bovine Parainfluenza-3 virus; *M. bovis*: *Mycoplasma bovis*

**Supplementary file 3. Table S3. Calf age at the beginning of treatment and weight gain from 14 days after beginning treatment to the 180^th^ day of life**

| **Parameters** | **Group of calves** | | | |
| --- | --- | --- | --- | --- |
|  | **EM**  **n=10** | **ES**  **n=10** | **CM**  **n=10** | **CS**  **n=10** |
| Mean age in days±SD at the start of treatment | 52±23,0 | 47,8±17,2 | 63,3±18,4 | 48,3±19,7 |
| Range of age in days at beginning of treatment (median) | 27-93  (49,5) | 24-76  (46,5) | 27-98  (65,5) | 19-80  (49,5) |
| Average daily gain±SD from 2 weeks after start of treatment to 6th month of life, (g/day) | 857±99 | 833±84 | 809±175 | 776±111 |
| Range of individual weight gains, (g/day) | 675-961 | 658-970 | 530-1085 | 562-938 |

Legend: EM: group treated with serum with moderate signs of respiratory disease; ES: group treated with serum with severe signs of respiratory disease; CM: group treated without serum with moderate signs of respiratory disease; CS= group treated without serum with severe signs of respiratory disease
